# Direct evidence of host-mediated glycosylation of NleA and its dependence on interaction with the COPII complex

**DOI:** 10.1080/19490976.2024.2305477

**Published:** 2024-02-01

**Authors:** Lindsay Burns, François Le Mauff, Samantha Gruenheid

**Affiliations:** a Department of Microbiology and Immunology, McGill University, Montreal, QC, Canada; b Infectious Disease and Immunity in Global Health Program, Research Institute of the McGill University Health Centre, Montreal, QC, Canada; c Glyco-NET Integrated Services, Microbial Glycomic Node, Montreal, QC, Canada; d McGill Interdisciplinary Initiative in Infection and Immunity, Montreal, QC, Canada

**Keywords:** EPEC, EHEC, Citrobacter rodentium, type III secretion, effectors, NleA, post-translational modification, glycosylation, O-GalNAc, Tn antigen, core 1 O-glycan, COPII

## Abstract

Non-LEE-encoded Effector A (NleA) is a type III secreted effector protein of enterohaemorrhagic and enteropathogenic *Escherichia coli* as well as the related mouse pathogen *Citrobacter rodentium*. NleA translocation into host cells is essential for virulence. We previously published several lines of evidence indicating that NleA is modified by host-mediated mucin-type O-linked glycosylation, the first example of a bacterial effector protein modified in this way. In this study, we use lectins to provide direct evidence for the modification of NleA by O-linked glycosylation and determine that the interaction of NleA with the COPII complex is necessary for this modification to occur.

## Introduction

Enteropathogenic *Escherichia coli* (EPEC) and enterohaemorrhagic *E. coli* (EHEC) are foodborne gastrointestinal pathogens that account for significant morbidity and mortality worldwide. EPEC infection can cause profuse watery diarrhea and is a primary cause of mortality in children under 5 years old.^1^ Those afflicted with EHEC infection also suffer from diarrhea, which in some cases can progress to life-threatening hemolytic uremic syndrome. EPEC, EHEC, and the closely related mouse pathogen *Citrobacter rodentium* are aptly named attaching and effacing pathogens due to their distinct ability to tightly adhere to the host epithelium, efface the absorptive microvilli architecture, and rearrange the cytoskeleton resulting in the formation pedestal-like lesions beneath attached bacteria.^2–4^ Development of A/E lesions is conferred by genes encoded in the Locus for Enterocyte Effacement (LEE)^5–7^ which also encodes a type III secretion system (T3SS) that is essential for pathogenicity.^8,9^ EPEC and EHEC utilize the T3SS to inject effector proteins that subvert and modulate host processes to promote bacterial survival and replication within the host.

Continued identification of novel effectors has shown that the effector repertoire of A/E pathogens is much larger than previously thought, with a complex functional network. Although the LEE encodes a small number of effector proteins, effectors encoded outside the LEE can also be translocated by the T3SS. Non-LEE-encoded effector A (NleA; also called EspI) is one of the first identified T3SS-translocated effectors located within a pathogenicity island separate from the LEE.^10^ NleA is absent from strains of nonpathogenic *E. coli* but preferentially found in strains associated with human disease.^11,12^ Although NleA’s function has been strongly implicated in bacterial virulence *in vivo*,^10,13,14^ the mechanism underlying its strong effect during infection is not well characterized. In our recent publication, we noted an increase in apparent molecular weight of NleA upon its translocation into host cells and presented several lines of evidence that this mobility shift is specifically due to host-mediated mucin-type O-linked glycosylation of NleA.^15^ Treatment of cells with brefeldin A and monensin, which inhibit Golgi trafficking, limited the apparent size shift of the NleA protein, implicating a role for the host cell secretory pathway in the modification of NleA.

The Golgi apparatus is the primary site of O-glycosylation and functions as the main sorting hub of the eukaryotic secretory pathway. Transport of proteins along this pathway begins at the endoplasmic reticulum (ER), where proteins are co-translationally translocated into the ER to undergo folding, modification, and quality control measures. After, proteins are segregated and exported from the ER via coat protein complex II (COPII) transport vesicles to either the ER-Golgi intermediate compartment or Golgi for further targeting to their ultimate intra- or extracellular destinations. Notably, NleA has previously been shown to localize to the secretory pathway, colocalizing with markers of the Golgi apparatus^10,16,17^ and the COPII complex has been identified as a binding partner of NleA.^18,19^ NleA possesses no known Golgi targeting motifs, but a site-directed mutagenesis study of NleA identified a complex motif on NleA that when mutated, completely abrogated Sec24 binding (NleA_DMΔIIQ_).^20^ Although this mutant was shown to be properly folded, it did not localize to the Golgi, and did not confer virulence in an *in vivo* mouse model.

In this addendum, we build on our previously published results to provide direct evidence for O-linked glycosylation of NleA and, using the non-Sec24-binding mutant, implicate COPII interaction as necessary for modification of NleA.

## Results

### NleA is O-glycosylated

**Figure 1.**
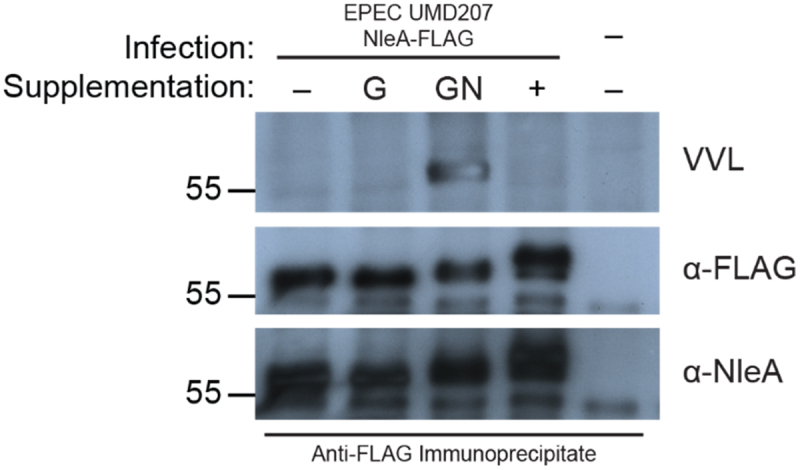
NleA is modified by O-linked glycosylation. Western blot analysis of immunoprecipitate from CHO ldlD cells infected with EPEC UMD207 NleA-FLAG probed with *vicia villosa* lectin (VVL), and anti-FLAG, and anti-NleA antibodies. Cells were cultured in media without (-), with galactose (G), with GalNAc (GN), or with both galactose and GalNAc (+) sugar supplementation. Migration of molecular weight markers (kDa) is indicated on the left.

Previous work has determined that the post-translational modification of NleA consisted of O-glycan additions to residues located between amino acid 169 and 183 and was not attributable to N-linked glycosylation, ubiquitination, or phosphorylation.^15^ To obtain further evidence of NleA’s host-mediated O-glycosylation, we infected Chinese Hamster Ovary (CHO) ldlD cells. These cells lack the UDP-Gal/UDP-GalNAc 4-epimerase enzyme, preventing the synthesis of the O-glycan precursors UDP-galactose and UDP-N-acetylgalactosamine (UDP-GalNAc).^21^ However, sugar salvage pathways allow the cell to uptake galactose and GalNAc from the environment, restoring O-glycosylation mechanisms.^22^ As we previously showed, when non-supplemented CHO ldlD cells were infected with EPEC UMD207 expressing FLAG-tagged NleA, the apparent molecular weight of NleA was slightly above 55kDa. In contrast, the apparent molecular weight of NleA increased only when the cells were supplemented with GalNAc, and was further increased when GalNAc and galactose were supplemented (Figure 1, middle and bottom panels). The requirement of GalNAc for the mobility shift of NleA and its extension post-galactose supplementation implicates that NleA acquired mucin-type O-linked glycosylation under the form of Tn antigens extending further to core-1 O-glycans, or T antigen.^22^

Next, we used lectins, proteins that bind specific glycans, to probe immunoprecipitated NleA from the conditions described above to directly assess the presence of carbohydrate additions on NleA. When probed with *Vicia villosa* lectin (VVL), which preferentially binds to a single α- or β-linked terminal GalNAc, a prominent band at the same size as NleA was revealed in the sample supplemented with GalNAc alone (Figure 1, top panel). Immunoprecipated NleA protein from cells without sugar supplementation or with only galactose had no discernible bands when probed with VVL (Figure 1). Interestingly, when galactose was used in combination with GalNAc, the VVL signal previously observed disappeared (Figure 1). Given that VVL binds only terminal GalNAc residues, the absence of signal with the dual complementation Gal+GalNAc therefore suggests that NleA O-glycosylation extends further than the Tn antigen toward a core-1 O-glycan structure which cannot be recognized by VVL.^23^

No signal was observed when probed with lectins recognizing other core glycans (data not shown). Thus, we have obtained direct evidence of the initial addition of GalNAc to NleA, implicating mucin-type O-linked glycosylation as the host-mediated modification. Furthermore, the loss of VVL reactivity, combined with the additional increase in apparent molecular weight when both sugars are supplemented, strongly suggests that the Tn antigens are further processed in the Golgi apparatus creating core-1-based O-glycan structures.

### Modification of NleA is dependent on binding to Sec24

**Figure 2.**
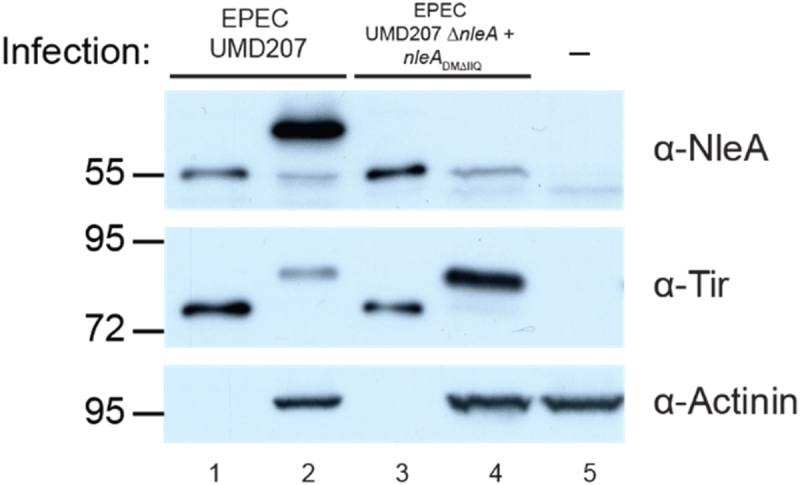
NleA deficient in Sec24 binding does not get modified. Western blot analysis of HeLa cell lysate infected with EPEC UMD207 expressing WT NleA (lane 2), NleA_DMΔIIQ_ (lane 4), or uninfected (lane 5). Bacterial lysates from the indicated strain are present in lanes 1 and 3. Blots were probed with anti-NleA, anti-tir, and anti-actinin antibodies. Migration of molecular weight makers (kDa) is indicated on the left.

Mucin-type O-linked glycosylation occurs within the secretory pathway of host cells.^24^ Considering that NleA localizes to the secretory pathway and binds the Sec24 component of the COPII vesicle coat, we took advantage of a mutant NleA_DMΔIIQ_ protein previously described to have significantly diminished interaction with Sec24 and loss of localization to the secretory pathway^20^ to assess whether this impacted the host-mediated modification of NleA. HeLa cells were infected with bacteria expressing WT NleA or NleA_DMΔIIQ_ and the apparent molecular weight of NleA was analyzed by Western blot (Figure 2). As expected, WT NleA displayed a significant mobility shift from its bacterial lysate size of 55 kDa (lane 1) to approximately 60–65 kDa in infected host cell lysates (lane 2). However, no such shift was seen in cells infected with bacteria expressing NleA_DMΔIIQ_, where the bacterial and host-associated NleA protein was visible at 55 kDa (lane 3 and 4, respectively). As a control for bacterial protein translocation, blots were assessed for another T3SS-translocated protein, Tir, which is known to undergo a mobility shift due to host-mediated phosphorylation, and the host protein α-actinin was assessed as a control for equal loading of cell lysates. Together, these data indicate that NleA’s interaction with the Sec24 component of the COPII complex seems to be important for its host-mediated post-translational modification to occur.

## Concluding remarks

In this addendum, we have built on our previous findings which indirectly implicated mucin-type O-linked glycosylation in the host-mediated modification of NleA by now providing direct evidence for this modification. Given the pattern of lectin binding to NleA when cells were cultured in media supplemented with both galactose and GalNAc, as well as the size of the mobility shift, we suspect that the final glycan addition on NleA is more complex and more extensive than the Tn antigen form of a single carbohydrate. Future work could narrow down which amino acids are substituted, and which glycans are present using single amino acid replacement of highly predicted sites and mass spectrometry technologies.

One question that remains is how NleA, an exogenous protein that is translocated into host cells fully translated, encounters the O-glycan synthetic machinery involving at least polypeptide GalNAc-transferases, C1GalT-1 and Cosmc.^24–26^ The interaction of NleA with host secretory pathway proteins was not investigated here, as we and others have identified only Sec23 and Sec24 as NleA binding partners of the COPII complex.^18,27^ Given that we showed NleA binding Sec24 is upstream of its host-mediated glycosylation, we might propose that the NleA-Sec24 interaction could aid in its localization to the secretory pathway via the anterograde trafficking vesicles. However, it is also possible that NleA follows a path of eukaryotic proteins known to enter the ER via post-translational translocation. Although eukaryotic proteins are typically translocated into or across the ER membrane co-translationally, several pathways for post-translational translocation have been described (reviewed in.^28^ In this process, specific fully synthesized proteins are transported from the cytoplasm into the lumen of the ER via accessory proteins that feed a polypeptide chain into the pore and drive translocation. It may not be surprising, then, that NleA is secreted into the cytoplasm of the host cell where it is trafficked to the protein secretory pathway, despite NleA having no identified host chaperone-binding partners or Golgi-targeting motifs.^10^ While NleA signal has been observed to colocalize with markers of the secretory pathway during infection^10,16,17^, NleA_DMΔIIQ_ instead showed diffuse, sustained cytosolic localization within the cell.^20^ Therefore, Sec24 binding is important for NleA modification and localization, but we cannot be sure which event precedes the other. Future studies are warranted to further dissect trafficking and localization of NleA over the course of infection to determine a timeline and trajectory of its pathway through the host cell.

*In vivo* studies demonstrate that NleA has an essential role in bacterial virulence. However, the mechanism by which NleA provokes disease, and the involvement of its O-linked glycosylation in this function, is not fully clear. We have previously shown that *C. rodentium* expressing a non-modifiable NleA mutant were indistinguishable from WT in an acute mortality mouse model, but did have a modest increase in persistence in a mixed infection model.^15^ Thus, the consequence of the host-mediated modification of NleA may be more relevant to host response to infection rather than to bacterial pathogenicity. We anticipate that future studies will provide clarity on the significance of NleA O-glycosylation on host immune modulation.

Overall, the results in this addendum augment our understanding of the interaction between NleA and host during infection, providing additional evidence of the protein secretory pathway as the locale for its host-mediated O-linked glycosylation.

## Data Availability

The data that support the findings of this study are available from the corresponding author, SG, upon reasonable request.
